# Complications of Robotic Pelvic Lymph Node Dissection for Prostate Cancer: An Analysis of the National Surgical Quality Improvement Program Targeted Prostatectomy Database

**DOI:** 10.3390/curroncol32110642

**Published:** 2025-11-16

**Authors:** Vatsala Mundra, Renil S. Titus, Eusebio Luna-Velasquez, Jiaqiong Xu, Carlos Riveros, Sanjana Ranganathan, Aamuktha Porika, Brian J. Miles, Dharam Kaushik, Christopher J. D. Wallis, Raj Satkunasivam

**Affiliations:** 1 Department of Urology, Houston Methodist Hospital, Houston, TX 77030, USA; 2 Center for Health Data Science and Analytics, Houston Methodist Research Institute, Houston, TX 77030, USA; 3 Division of Urology and Surgical Oncology, Department of Surgery, Princess Margaret Cancer Centre, University Health Network, University of Toronto, Toronto, ON M5S 1A1, Canada; 4 Division of Urology, University of Toronto, Toronto, ON M5S 1A1, Canada; 5 Division of Urology, Mount Sinai Hospital, Toronto, ON M5G 1X5, Canada

**Keywords:** prostate cancer, lymph node, radical prostatectomy, robotic

## Abstract

Introduction/Background: Treatment of localized prostate cancer includes radical prostatectomy (RP) with or without pelvic lymph node dissection (PLND). While multiple guidelines recommend PLND for staging purposes, recent data has shown questionable therapeutic benefit. Thus, understanding the morbidity associated with PLND is important for counseling patients. We used the American College of Surgeons National Surgical Quality Improvement Program (ACS NSQIP) targeted prostatectomy database to quantify real-world 30-day postoperative outcomes of patients undergoing contemporary robot-assisted PLND at the time of RP for prostate cancer to quantify the incremental morbidity. Methods: We conducted a retrospective cohort study using the NSQIP database of adult patients undergoing radical prostatectomy from 2019 to 2022. The primary outcomes were procedure-specific outcomes such as lymphocele and rectal injury. Secondary outcomes included a composite of any of the following 30-day major postoperative outcomes: mortality, reoperation, cardiac or neurologic event, as well as the individual components of this outcome, as well as infectious and other complications. We also analyzed yearly trends associated with PLND. Groups were balanced using propensity score matching (PSM) with a 1:1 ratio using demographic characteristics, prior medical history, and cancer staging data. Likelihood of complications was assessed by conditional logistic regression. Results: We identified 13,413 patients between 2019 and 2022 who underwent robotic prostatectomy: 11,341 (85%) had PLND while 2072 (15%) did not. After PSM, our cohort included 2071 matched pairs of patients with and without PLND. Patients who underwent PLND were more likely to be diagnosed with lymphocele (2.14% vs. 0.68%, OR 4.17; 95% CI 2.00, 8.68), have unplanned readmission (4.22% vs. 3.27%, OR 1.31; 95% CI 1.03, 1.65), and develop organ-site/space SSI (1.18% vs. 0.60%) (OR 1.97, 95% CI 1.20, 3.23). There was no significant association between the receipt of PLND and the likelihood of urinary leak or fistula, or ureteral obstruction. There were no significant differences between the two groups with respect to secondary outcomes of interest. Conclusion: Contemporary robotic PLND is associated with a 3-fold increased likelihood of lymphocele, as well as increased likelihood of unplanned readmission and organ-site SSI, though no significant differences in major postoperative complications were identified. We found that the odds of lymphoceles, readmission, and SSI in our study are lower than previously reported. These data provide real-world data to guide patient counseling and optimize patient selection for PLND at the time of RALP.

## Simple Summary

Prostate cancer is often treated with surgery to remove the prostate, sometimes along with nearby lymph nodes, to check if the cancer has spread. While removing these lymph nodes helps with cancer staging, it is uncertain whether the procedure improves survival, and it may carry additional risks. Using a large national database, we compared patients who had prostate surgery with and without lymph node removal to understand the added risks. We found that those who had lymph node removal had a small risk of developing fluid collections (lymphoceles), surgical site infections, and requiring hospital readmission, but they did not experience higher rates of serious complications. These findings suggest that while lymph node removal provides staging information, it adds small short-term risks. This information can help doctors and patients make better decisions about when lymph node removal is truly necessary.

## 1. Introduction

Prostate cancer is the second most common cancer in men worldwide and accounts for 3.8% of all cancer deaths in men. The standard of care for localized prostate cancer is either radiation therapy or radical prostatectomy (RP) with or without pelvic lymph node dissection (PLND). Multiple guidelines recommend PLND for staging purposes and there may also be a therapeutic benefit from removing low-volume metastatic disease [1,2]. However, PLND is not without complications and prior studies have shown an association with an increased operative time, lymphoceles, increased rate of blood transfusions, deep vein thrombosis (DVT), and pulmonary embolism (PE) [3,4,5,6,7,8]. Multiple strategies have been employed to limit these risks, including the use of predictive nomograms to optimize patient selection and novel surgical techniques, including Lahey peritoneal advancement, use of tissue sealant products, clips, and energy devices [9,10]. Despite these developments, there remains a paucity of real-world data assessing the morbidity of contemporary robot-assisted PLND.

We therefore sought to use the American College of Surgeons National Surgical Quality Improvement Program (ACS NSQIP) targeted prostatectomy database to quantify trends associated with PLND and the rates of real-world 30-day postoperative outcomes of patients undergoing PLND at the time of RP for prostate cancer and quantify the incremental morbidity by comparing to those who underwent RP without PLND. The database provides population-based granular data for prostatectomy-specific complications such as lymphoceles and urinary leak as well as other conventional 30-day postoperative complications.

## 2. Methods

### 2.1. Study Design and Population

Our retrospective cohort study utilized the American College of Surgeons National Surgical Quality Improvement Program (ACS NSQIP) database, which collects standardized 30-day postoperative outcomes from more than 700 participating hospitals across the United States. These hospitals include large academic centers, tertiary referral institutions, and community hospitals, providing a broad and nationally representative sample of contemporary surgical practice. Since 2019, ACS NSQIP has also developed targeted procedure-specific datasets, including the prostatectomy database, which allow for improved granularity in operative details and complication tracking. We used the 2019–2022 targeted prostatectomy dataset to evaluate adverse outcomes associated with pelvic lymph node dissection (PLND).

### 2.2. Inclusion and Exclusion Criteria

All adult patients (age ≥ 18) who were diagnosed with prostate cancer (International Classification of Diseases (ICD) code C61x) and underwent robotic prostatectomy were included. Exclusion criteria included patients that were above the age of 90, non-elective cases, renal failure, metastatic disease, ventilator dependence, infection at time of surgery, and ASA score of 5. We also excluded patients that were missing any of the following information: height, weight, functional health status, and length of hospital stay (LOS).

### 2.3. Exposure

The exposure was whether a PLND was completed at the time of robotic radical prostatectomy. We operationalized this by evaluating the number of nodes removed, with receipt of PLND defined as any node count including and above one.

### 2.4. Primary Outcome

The primary outcome was procedure-specific complications which included rectal injury, urinary leak or fistula, and lymphocele or other lymphatic leak (a postoperative fluid collection that is not identified as an abscess, GI leak, or urine leak).

### 2.5. Secondary Outcomes

Secondary outcomes included the number of nodes removed in the PLND cohort and PLND trends from 2019 to 2022. The secondary outcome was a composite of major complications. Other outcomes included individual components of each of the secondary complications as well as general surgical complications, including reintubation, ventilator dependence > 48 h, sepsis, urinary tract infection (UTI), surgical site infection (SSI) (superficial, deep, and organ site), pulmonary embolism (PE), deep vein thrombosis (DVT), prolonged LOS (defined as > 1 day, which was the median LOS in our cohort), occurrence of transfusion within 72 h from surgery, length of stay > 30 days, wound dehiscence, unplanned readmission, acute renal failure, and *Clostridium difficile* infection.

### 2.6. Covariates

Covariates in this study were chosen according to clinical relevance to postoperative outcomes in patients undergoing robotic radical prostatectomy. We included demographic and clinical characteristics such as age, race, ethnicity, body mass index (BMI), smoking status, ASA score, chronic steroid use, modified frailty index (MFI-5), pathologic T stage, and stent placement.

### 2.7. Statistical Analysis

Data were represented as mean ± standard deviation (SD) for continuous variables, median and interquartile range for skewed continuous variables, and number (%) for categorical variables. Propensity score matching (PSM) was used to minimize differences between the two cohorts: those who underwent PLND and those who did not. PSM was conducted using a multivariable logistic regression model with the dependent variable being the type of treatment while the independent variables were the aforementioned covariates. We used a 1:1 ratio between PLND and no PLND through a greedy algorithm based on a caliper of 0.01 to balance the covariates between the PLND and non-PLND group [11]. After PSM, all baseline characteristics were balanced between the two groups for an absolute standardized mean difference of <0.1. To assess for heterogeneity of effect in the regression model, we looked at interaction terms between operative approach and a priori defined subgroups: age (greater than or less than 65 years old), BMI, prior pelvic operations, and node count. Statistical analyses were conducted using STATA version 17 (StataCorp. 2021. Stata Statistical Software: Release 17. College Station, TX, USA: StataCorp LLC). A two-tailed *p*-value threshold of 0.05 was considered statistically significant for all comparisons.

## 3. Results

A total of 13,413 patients met the inclusion criteria. We found statistically significant differences in age, race, Hispanic ethnicity, pathologic T stage, and drain placement between those who underwent PLND and those who did not. Patients undergoing PLND were more likely to be white (73.3% vs. 68.9%) and non-Hispanic (89.0% vs. 84.3%). A higher proportion of patients receiving PLND had a pathological T stage between T3 and T4 relative to patients not receiving PLND (45.7% vs. 29.3%). After PSM, a total of 4142 patients were in the cohort: 2071 patients were in the No PLND cohort and 2071 were in the PLND cohort. Covariates were balanced as indicated by an SMD of <0.1 (Table 1).

Overall, the proportion of patients receiving PLND at the time of RALP increased over time, though of debatable clinical importance: from 84.23% (n = 5898) in 2019 to 85.87% (n = 5786) by 2022 (Table 2). In the PLND cohort, the median number of nodes removed was 6 (IQR 3–11) (Table 2).

### 3.1. Primary Outcome

We observed an increased likelihood of lymphocele among patients undergoing PLND, with a crude rate of lymphocele of 2.14% in the PLND cohort and 0.68% in those who did not receive PLND. Receipt of PLND was associated with an increased risk of lymphoceles (OR 3.20, 95% CI 2.06–4.95). We did not identify that the receipt of PLND was associated with an increased risk of prostatectomy-specific complications such as rectal injury, urinary leak, anastomotic leak, and prolonged postoperative NPO or NG tube (Table 3).

### 3.2. Secondary Outcomes

We failed to identify any differences in 30-day mortality, return to OR, MI, and stroke/CVA among patients who received PLND compared to those that did not. We did observe a statistically significant difference in unplanned readmission as well as organ-site SSI. Those undergoing PLND had a crude rate of 4.22% of having a readmission while those not undergoing PLND had a rate of 3.27% (OR 1.31, 95% CI 1.03–1.65). In addition, those undergoing PLND had an increased likelihood of developing an organ-site SSI: the crude rate of developing an SSI in the PLND cohort was 1.18% while in the non-PLND cohort it was 0.60% (OR 1.97, 95% CI 1.20–3.23). On the other hand, patients that did not receive a PLND were associated with a greater risk of superficial SSI compared to those that did receive a PLND (0.60% vs. 0.25%, OR 0.42, 95% CI 0.20–0.87). These findings did not translate to differences in sepsis between patients that received PLND (0.65%) as compared to those that did not (0.43%) (OR 1.53, 95% CI 0.83–2.83) (Table 3).

### 3.3. Subgroup Analysis

To assess for heterogeneity of effect, we conducted subgroup analyses examining the receipt of PLND and the primary outcomes of interest, stratified by age (<65 vs. ≥65) and body mass index (BMI, <25 vs. ≥25). We found that regardless of age and BMI, receipt of PLND was associated with an increased likelihood of developing a lymphocele (Table 4).

## 4. Discussion

We performed a contemporary retrospective cohort study using the ACS-NSQIP dataset with prostatectomy targeted variables to analyze the trend in the use of PLND at the time of RALP, and 30-day morbidity associated with this procedure. Over 2019–2022, patients undergoing PLND at the time of robotic radical prostatectomy were at 3-fold increased likelihood of lymphoceles, 1.3-fold increased likelihood for unplanned readmission, and 2-fold increased odds of organ-site SSI relative to those that did not have PLND. We also found that not having a PLND was associated with a 2.4-fold increased odds of superficial SSI.

Our findings were consistent with the literature in that there is an increased likelihood of adverse events in patients who have PLND. Previous studies have shown that the odds of lymphoceles are inherently associated with PLND [12,13,14,15,16]. For example, an international prospective study from 2006 to 2009 found that patients undergoing both limited (OR 12.50) and extended PLNDs (OR 17.24) had a higher likelihood of developing pelvic lymphocele relative to those that did not receive a PLND (*p* < 0.001). When examining a single surgeon at a single institution cohort from 2007 to 2011, Liss et al. found an increased odds of lymphoceles (*p* = 0.011) with both an extended PLND (5.6%) and standard PLND (2.2%) when compared to no PLND (0%) [12]. Two North American studies examining lymphocele following RALP found higher odds in those undergoing a PLND, but not a level of statistical significance [6,17]. While we identified a statistically significant difference between the association of PLND and lymphoceles, the absolute value (2.14%) was lower than in comparison to the aforementioned studies.

As observed in our study, the previous literature has also found that PLND has been associated with higher rates of readmission, specifically for occluded catheters, infections, and lymphoceles [18]. Infectious complications and catheter-related issues are common to all patients undergoing RP; however, the increase in readmission rates in those who have a PLND is likely due to lymphoceles and other complications unique to receiving the PLND. Furthermore, our NSQIP-based study also found an increase in superficial-SSIs in those who did not have a PLND. This is perhaps due to comorbidities that inhibit the patient from receiving a PLND and increase their likelihood of infection. Conversely, we found that those undergoing PLND were associated with higher odds of organ-site SSI. Omitting PLND may reduce deep dissection, which could explain the lower odds of organ-site SSI [19].

Sepsis is not a commonly reported complication in regard to PLND. In a 2015 meta-analysis looking at 66 studies, only one reported on sepsis as a complication of PLND [3]. In this study, Lindberg et al. performed a single-institution retrospective study from 2002 to 2007 looking at the difference in complications between extended PLNDs (ePLND) and limited PLNDs (lPLND) and found a higher associated likelihood of sepsis for patients undergoing ePLNDs (3%) than lPLND (0%) [20]. They do not specify what type of sepsis the patients experienced. Furthermore, the rate of organ-site SSI associated with PLND that we observed was much lower than the 3% reported by Lindberg et al. This may be due to our cohort being composed of only robotic prostatectomies which have been shown to decrease SSI rates relative to open surgery [21].

In addition, other adverse outcomes commonly studied in association with PLND, such as increased operative time, prolonged length of hospital stay, and higher likelihood of mortality, were not significant in our study. Our results of lower rates of lymphocele and DVT than in the current literature and other nonsignificant adverse outcomes can be explained by a variety of factors. First, our study only looked at robotic prostatectomy cases which, when compared to open, have been shown to have improved postsurgical complications [22,23]. Furthermore, increased surgeon experience is correlated with better outcomes, and with advances in imaging techniques, such as multiparametric magnetic resonance imaging (mpMRI), patient selection is further optimized [24,25].

There are limitations to our retrospective study. While we used PSM to decrease the inherent bias, there may still be other unmeasured biases such as surgeon or facility level bias. Furthermore, there may be confounders such as patient selection that impact treatment and outcomes that are difficult to measure in our study. In addition, we are only able to analyze 4 years of data as the targeted prostatectomy database was initiated in 2019 by NSQIP.

Although the oncological benefits of PLND are debated, there is a consensus that PLND is necessary for staging as it determines further management. Other national multicenter studies showed that there was a steady uptrend of usage of PLND [26,27]. Pre-surgical imaging is not sensitive enough to accurately stage: mpMRI has a sensitivity of less than 40% in detecting lymph node invasion in prostate cancer [1]. And, while prostate-specific membrane antigen positron emission tomography is better with a sensitivity of 49–66%, it is still not ideal [26]. For these reasons, PLND is the preferred staging technique and is imperative in determining treatment and prognosis. While our study does not capture surgical technique with sufficient granularity, several intraoperative strategies have been shown to reduce PLND-associated morbidity, such as peritoneal flap creation to promote intraperitoneal drainage of lymphatic fluid as a strategy to reduce lymphoceles. Whether PLND meaningfully influences postoperative quality of life has not been well established; prospective studies with standardized patient-reported outcomes are needed to clarify this relationship.

Overall, our contemporary retrospective population-level analysis from 2019 to 2022 showed that patients undergoing robotic RP and PLND for prostate cancer had increased odds of lymphoceles and unplanned readmission relative to patients that had a robotic RP with no PLND. The likelihood for lymphoceles, unplanned readmission, and SSI was lower in our cohort than previously reported, and therefore, we conclude that PLND can be performed with a minimally increased likelihood in selected prostate cancer patients undergoing robotic RP.

## 5. Conclusions

Lymph node removal during prostate cancer surgery is associated with a small increased risk of certain short-term complications but does not appear to affect major outcomes. These findings highlight the importance of carefully selecting patients for this procedure to balance staging benefits against potential risks.

## Figures and Tables

**Table 1:** Baseline characteristics by PLND before propensity score matching (PSM).

	*Before PSM*			*After PSM*		
	No PLND	PLND	SMD	No PLND	PLND	SMD
	N = 3981	N = 22,461		N = 3981	N = 3981	
Age	62.94 ± 6.97	63.71 ± 6.76	−0.113	62.94 ± 6.97	63.17 ± 6.92	−0.033
Race			0.078			0.023
White	2644 (66.42)	15,518 (69.09)		2644 (66.42)	2654 (66.67)	
Black or African American	470 (11.81)	2750 (12.24)		470 (11.81)	491 (12.33)	
Other/Unknown	867 (21.78)	4193 (18.67)		867 (21.78)	836 (21.00)	
Hispanic			0.104			0.012
No	3144 (78.98)	18,583 (82.73)		3144 (78.98)	3160 (79.38)	
Yes	186 (4.67)	1017 (4.53)		186 (4.67)	178 (4.47)	
Unknown	651 (16.35)	2861 (12.74)		651 (16.35)	643 (16.15)	
BMI	29.24 ± 4.78	29.18 ± 4.82	0.013	29.24 ± 4.78	29.13 ± 4.79	0.023
Current Smoker			0.005			0.006
No	3577 (89.85)	20,146 (89.69)		3577 (89.85)	3570 (89.68)	
Yes	404 (10.15)	2315 (10.31)		404 (10.15)	411 (10.32)	
ASA			0.025			0.004
1–2	2164 (54.36)	11,931 (53.12)		2164 (54.36)	2156 (54.16)	
3–4	1817 (45.64)	10,530 (46.88)		1817 (45.64)	1825 (45.84)	
Chronic steroid use			0.023			0.002
No	3911 (98.24)	21,996 (97.93)		3911 (98.24)	3912 (98.27)	
Yes	70 (1.76)	465 (2.07)		70 (1.76)	69 (1.73)	
Modified frailty index			0.022			0.029
Score 0–1	1794 (45.06)	9877 (43.97)		1794 (45.06)	1736 (43.61)	
Score 2+	2187 (54.94)	12,584 (56.03)		2187 (54.94)	2245 (56.39)	
Pathologic T stage			0.338			0.065
T0–T2	2771 (69.61)	11,987 (53.37)		2771 (69.61)	2651 (66.59)	
T3–T4	1210 (30.39)	10,474 (46.63)		1210 (30.39)	1330 (33.41)	
Stents			0.068			0.036
No	3848 (96.66)	21,412 (95.33)		3848 (96.66)	3821 (95.98)	
Yes	133 (3.34)	1049 (4.67)		133 (3.34)	160 (4.02)	
Drains			0.162			0.054
No	2155 (54.13)	10,344 (46.05)		2155 (54.13)	2047 (51.42)	
Yes	1826 (45.87)	12,117 (53.95)		1826 (45.87)	1934 (48.58)	
Total operation time	194 (152–236)	201 (158–248)	−0.092	194 (152–236)	195 (154–241)	−0.004

**Table 2:** PLND by year before PSM.

	No PLND	PLND	Median Nodes Removed
	N (%)	N (%)	Median (IQR)
Year of Operation			
2019	1104 (15.77)	5898 (84.23)	7 (4–12)
2020	909 (14.93)	5180 (85.07)	7 (3–11)
2021	1016 (15.36)	5597 (84.64)	6 (3–11)
2022	952 (14.13)	5786 (85.87)	6 (3–11)
Overall	3981	22,461	6 (3–11)

Number of PLNDs completed by year (*p* = 0.019) and the median number of nodes removed by year.

**Table 3:** Outcomes by PLND after PSM.

	*Total*	*No PLND*	*PLND*	*OR (95% CI) (PLND vs. No PLND)*	*p-Value*
	N = 7962	N = 3981	N = 3981		
Prostatectomy-specific Complications					
Rectal injury	22 (0.28)	13 (0.33)	9 (0.23)	0.69 (0.29, 1.62)	0.396
Urinary leak or fistula	104 (1.31)	55 (1.38)	49 (1.23)	0.89 (0.60, 1.31)	0.557
Ureteral obstruction	31 (0.39)	13 (0.33)	18 (0.45)	1.39 (0.68, 2.84)	0.371
Anastomotic leak	467 (5.87)	241 (6.05)	226 (5.68)	0.93 (0.77, 1.13)	0.481
Lymphocele or other lymphatic leak	112 (1.41)	27 (0.68)	85 (2.14)	3.20 (2.06, 4.95)	<0.001
Prolonged postop NPO or NGT use	64 (0.80)	31 (0.78)	33 (0.83)	1.07 (0.65, 1.73)	0.800
Major complications	103 (1.29)	48 (1.21)	55 (1.38)	1.15 (0.78, 1.68)	0.478
30 day mortality	10 (0.13)	5 (0.13)	5 (0.13)	1.00 (0.29, 3.46)	1.000
Returnor	80 (1.00)	35 (0.88)	45 (1.13)	1.29 (0.84, 1.98)	0.246
Cardiac arrest	6 (0.08)	4 (0.10)	2 (0.05)	0.50 (0.09, 2.73)	0.424
MI	13 (0.16)	6 (0.15)	7 (0.18)	1.17 (0.39, 3.48)	0.782
Stroke/CVA	4 (0.05)	2 (0.05)	2 (0.05)	1.00 (0.14, 7.11)	1.000
Unplanned Intubation	6 (0.08)	3 (0.08)	3 (0.08)	1.00 (0.20, 4.96)	1.000
Ventilator >48 h	6 (0.08)	1 (0.03)	5 (0.13)	5.01 (0.58, 42.89)	0.142
Sepsis	43 (0.54)	17 (0.43)	26 (0.65)	1.53 (0.83, 2.83)	0.173
Urinary Tract Infection	190 (2.39)	97 (2.44)	93 (2.34)	0.96 (0.72, 1.28)	0.769
Surgical site infection	109 (1.37)	50 (1.26)	59 (1.48)	1.18 (0.81, 1.73)	0.389
Superficial SSI	34 (0.43)	24 (0.60)	10 (0.25)	0.42 (0.20, 0.87)	0.020
Deep SSI	4 (0.05)	2 (0.05)	2 (0.05)	1.00 (0.14, 7.11)	1.000
Organ-Site SSI	71 (0.89)	24 (0.60)	47 (1.18)	1.97 (1.20, 3.23)	0.007
Pulmonary Embolism	39 (0.49)	20 (0.50)	19 (0.48)	0.95 (0.51, 1.79)	0.873
Deep vein thrombosis	22 (0.28)	10 (0.25)	12 (0.30)	1.20 (0.52, 2.79)	0.670
Prolonged LOS	1661 (20.86)	813 (20.42)	848 (21.30)	1.05 (0.95, 1.17)	0.325
Occurrence of Transfusion	161 (2.02)	78 (1.96)	83 (2.08)	1.07 (0.78, 1.45)	0.690
Still in hospital	1649 (20.71)	809 (20.32)	840 (21.10)	1.05 (0.94, 1.17)	0.382
Wound Disruption	7 (0.09)	2 (0.05)	5 (0.13)	2.50 (0.48, 12.91)	0.273
Unplanned readmission	298 (3.74)	130 (3.27)	168 (4.22)	1.31 (1.03, 1.65)	0.026
Acute Renal Failure	3 (0.04)	1 (0.03)	2 (0.05)	2.00 (0.18, 22.09)	0.571
C. diff	3 (0.04)	1 (0.03)	2 (0.05)	2.00 (0.18, 22.09)	0.571

**Table 4:** Subgroup Analysis.

	BMI				Age			
	<25		≥25		<65		≥65	
Outcomes	OR [95% CI]	*p*-Value	OR [95% CI]	*p*-Value	OR [95% CI]	*p*-Value	OR [95% CI]	*p*-Value
Rectal injury	NA		0.91 (0.37, 2.25)	0.84	1.69 (0.40, 7.08)	0.474	0.39 (0.12, 1.25)	0.115
Urinary leak or fistula	0.59 (0.19, 1.80)	0.351	0.95 (0.62, 1.44)	0.8	1.18 (0.68, 2.05)	0.549	0.66 (0.38, 1.16)	0.151
Ureteral obstruction	0.94 (0.13, 6.73)	0.952	1.48 (0.68, 3.19)	0.322	1.39 (0.56, 3.48)	0.476	1.38 (0.44, 4.36)	0.583
Anastomotic leak	0.84 (0.54, 1.33)	0.464	0.95 (0.77, 1.18)	0.66	0.95 (0.74, 1.24)	0.717	0.91 (0.69, 1.20)	0.505
Lymphocele or other lymphatic leak	3.81 (1.07, 13.58)	0.039	3.13 (1.96, 4.98)	<0.001	3.68 (2.02, 6.68)	<0.001	2.68 (1.41, 5.11)	0.003
Prolonged postop NPO or NGT use	0.94 (0.23, 3.79)	0.932	1.09 (0.65, 1.83)	0.748	1.01 (0.49, 2.08)	0.973	1.11 (0.57, 2.14)	0.758

## Data Availability

Data for this study was queried from publicly available database.
